# EEG Signals Analysis Using Multiscale Entropy for Depth of Anesthesia Monitoring during Surgery through Artificial Neural Networks

**DOI:** 10.1155/2015/232381

**Published:** 2015-09-28

**Authors:** Quan Liu, Yi-Feng Chen, Shou-Zen Fan, Maysam F. Abbod, Jiann-Shing Shieh

**Affiliations:** ^1^Key Laboratory of Fiber Optic Sensing Technology and Information Processing, Ministry of Education, Wuhan University of Technology, Wuhan, Hubei 430070, China; ^2^School of Information Engineering, Wuhan University of Technology, Wuhan, Hubei 430070, China; ^3^Department of Anesthesiology, College of Medicine, National Taiwan University, Taipei 100, Taiwan; ^4^College of Engineering, Design and Physical Sciences, Brunel University London, Uxbridge UB8 3PH, UK; ^5^Department of Mechanical Engineering and Innovation Center for Big Data and Digital Convergence, Yuan Ze University, Taoyuan, Chung-Li 32003, Taiwan

## Abstract

In order to build a reliable index to monitor the depth of anesthesia (DOA), many algorithms have been proposed in recent years, one of which is sample entropy (SampEn), a commonly used and important tool to measure the regularity of data series. However, SampEn only estimates the complexity of signals on one time scale. In this study, a new approach is introduced using multiscale entropy (MSE) considering the structure information over different time scales. The entropy values over different time scales calculated through MSE are applied as the input data to train an artificial neural network (ANN) model using bispectral index (BIS) or expert assessment of conscious level (EACL) as the target. To test the performance of the new index's sensitivity to artifacts, we compared the results before and after filtration by multivariate empirical mode decomposition (MEMD). The new approach via ANN is utilized in real EEG signals collected from 26 patients before and after filtering by MEMD, respectively; the results show that is a higher correlation between index from the proposed approach and the gold standard compared with SampEn. Moreover, the proposed approach is more structurally robust to noise and artifacts which indicates that it can be used for monitoring the DOA more accurately.

## 1. Introduction

Anesthesia is an indispensable stage for doctors during surgery and in the intensive care environment, which enables the patients to undergo surgery to keep unconsciousness and lack of pain through suppressing response of nervous system to nonnoxious stimuli [1–3]. However, interaction of anesthetic drugs and central nervous system is very complex, so methodologies for assessment of DOA are controversial but very important in medical domain [4–6]. Monitoring the DOA is not only to determine the patients' states during surgery but also to further control the amount of anesthetic required for individuals to ensure high quality and safety of anesthesia with rapid recovery after operation. Therefore, the necessity to evaluate and optimize DOA monitoring is absolutely important not only for surgeons during surgery but also for patients' health after operation.

In traditional methods, measurement of DOA is implemented by analysis of signals collected from patients such as electrocardiogram (ECG), respiration (Resp), blood pressure (BP), and peripheral oxygen saturation (SpO_2_) which reflect the consciousness level of patients indirectly. However, these signals cannot estimate the DOA accurately and are easily disturbed by artifacts and noise. EEG signal and auditory evoked potential (AEP) based monitors are the internationally recognized anesthesia monitoring method in operation [7, 8]. In particular, the methods based on EEG for DOA evaluation have been developed rapidly. The EEG signals which reflect the brain's activities have been widely used for research and diagnosis, especially for measuring the awareness level of patients. EEG referring to brain's electrical activity is commonly recorded in a noninvasive approach, which provides an available tool to study the human brain for researchers and doctors [9]. It has been widely used for measuring consciousness level of patients in medical environment [10–12].

There are various methods based on EEG analysis applied to monitor DOA recently. The bispectral index (BIS) monitor introduced by Aspect Medical Systems, Inc., in 1994 [13–15] is widely used in the operation room for evaluating the DOA by analysis of EEG signals of patients during surgery. BIS monitor has been proved as a reliable system to measure the DOA except for several anaesthetic agents in many researches [16, 17]. However, the company that introduced the BIS monitor has not disclosed the detailed algorithms. In addition, entropy monitors developed by Datex-Ohmeda produce response entropy (RE) and state entropy (SE) to evaluate the irregularity in EEG signals for determining the DOA [18]. The algorithm applied in the Datex-Ohmeda entropy module calculates the RE and SE based on frequency domain approach called spectral entropy which is obtained by applying Shannon entropy to the power spectrum [19]. However, application of fast Fourier transform (FFT) to estimate power spectrum may miss the nonlinear and nonstationary properties of EEG signals. Although these two monitor systems are the most popular, there are limitations. Therefore, an open source and time domain based method taking the nonlinear and nonstationary properties of EEG signals into consideration is need for monitoring DOA during surgery robustly and accurately.

The approximate entropy (ApEn) [20] and SampEn [21] algorithms are two powerful approaches proposed in application of determining the complexity of any time series. And SampEn has been proved to perform better than ApEn for monitoring DOA of patients during surgery in previous studies [21–23]. Nevertheless, SampEn measures complexity of time series based on a single time scale so that it misses the features associated with signal structure. To overcome this problem, Costa et al. introduced an improved method named multiscale entropy (MSE) to analyze the complexity of biological signals over multiple time scales [24, 25]. The EEG reflects the summation of human brain's activity and contains the information about neuronal dynamics underlying high and low frequency [26, 27]. Therefore, MSE is appropriate for obtaining the dynamics features related to multiple time scales and has been widely used in analysis of EEG recordings [28–30]. Although MSE measure can explore the degree of complexity over different time scales, a single index which indicates the DOA of patients during surgery is needed by surgeons. However, we find that, on the one hand, many studies applied MSE to distinguish the complexity of EEG through plotting entropy values over different time scales overall without considering time [31, 32] or calculate entropy values on all time scales independently for monitoring DOA [30] which is too complicated for surgeons to determine patients' anesthesia level. On the other hand, in previous research [33], a single index was derived from entropy values based on appropriate scales by averaging the scale dependent entropies. The limitation of this method is that entropies related to each scale contribute unequally to measure the complexity and it is difficult to confirm the weights for each independent scale. Therefore, in this paper, a new index is obtained from MSE analysis by combining the independent entropies via ANN for measuring the DOA during surgery.

The ANN is an extremely important and useful algorithm in machine learning inspired by biological neural networks [34]. It can be used to adaptively and optimally estimate the weights and functions which are generally unknown in advance to depend on the input and target data by training, validating, and testing. Therefore, it has been widely used to solve many tasks for classification and regression analysis in biomedical engineering [35].

In this study, simulated EEG corrupted with EOG and EEG collected from patients with different consciousness level are analyzed by MSE to investigate the sensitivity to EOG and ability to distinguish the patients' states of entropies corresponding to each independent scale. Next, we apply the MSE method to real EEG recordings collected from patients during surgery. And then entropies and a gold standard are defined as input and target variables to train the ANN model. The outputs of ANN are severed as the new combined index for DOA monitoring. BIS as a commercial index has been approved by US Food and Drug Administration and most widely used in operation room during surgery and ICU to monitor DOA although it is not perfect. For example, intraoperative awareness can occur during general anesthesia with a small probability event even if BIS value is under 60 according to recent researches [15, 36, 37]. BIS is one of the technologies to accurately monitor the hypnotic effects of general anesthetics and sedatives based on EEG signals. However, the device is very expensive, and the details of the algorithms to calculate BIS index have not been disclosed. So it is necessary to create an open sources method for DOA monitoring accurately. In this paper, our aim is to create a new index which can accurately trace the change of consciousness level of patients like BIS; therefore, BIS is used as a gold standard of DOA. However, it would be more applicable and reasonable if there is a real gold standard of DOA as the target. So in comparison, “the state of anesthetic depth” called expert assessment of conscious level (EACL) [38] which is decided by five experienced anesthesiologists based on detailed recordings during surgery was used as the target to train ANN.

EEG signals are always corrupted by artifacts, such as EOG and EMG. Generally, the amplitude of EEG can be extremely less than artifacts, so techniques are needed to remove EEG contaminants for accurate analysis. In this paper, MEMD [39] based filter was used to remove artifacts from contaminated EEG signals. On the one hand, through comparison of performance of proposed method before and after filtering, we can indicate the robustness of proposed method to artifacts. On the other hand, MSE measures complexity of time series at different scales, and filter can enhance the features related to some scales for monitoring DOA more accurately. If we combine the MSE at these scale, the indicator would have higher performance to measure DOA. It is indicated that the index is less sensitive to noise and perform highly better than SampEn.

## 2. Materials and Methods

### 2.1. Data Sources and EEG Recordings

The one channel EEG signals tested in this study are collected from twenty-six patients through a forehead mounted sensor by MP60 system (Philip, IntelliVue MP60 BIS module). They aged from 23 to 72 years are accepting ear, nose, and throat (ENT) surgery with general anesthesia at the National Taiwan University Hospital (NTUH) of Taiwan when recording EEG. And the drugs administered for anesthesia induction and corresponding anesthetic technique are sevoflurane or desflurane for tracheal intubation of 18 patients, sevoflurane or desflurane for laryngeal mask airway (LMA) of 5 patients, and propofol for total intravenous anesthesia of 3 patients, respectively. The sampling rate of EEG is 125 Hz.

### 2.2. Data Preprocess

**Figure 1:**
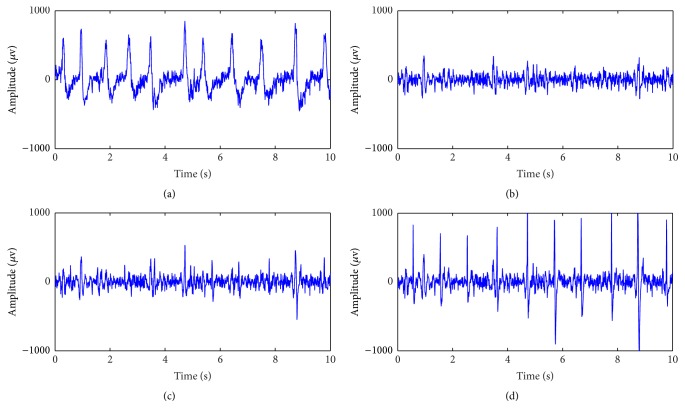
EEG signal under different conditions. (a) Original EEG collected from one patient under surgery. (b) EEG after filtering of (a) by summing IMF2 and IMF3 through MEMD. (c) EEG corrupted with 5 dB EOG of (b). (d) EEG corrupted with −5 dB EOG of (b).

According to the standard operation procedure (SOP) with general anesthesia, it can be divided into four stages, that is, the preoperation, induction, maintenance, and recovery [30]. The collected EEG are divided into three parts in this study due to different purposes. Firstly, we select ten patients at random to estimate the sensitivity of MSE from each independent scale to EOG noise. During the preoperation stage, patients prepare to accept the operation with consciousness and always blink their eyes frequently, so the EEG recordings during this stage are badly corrupted with EOG artifact as shown in Figure 1(a). The EEG signals during preoperation stage are collected from these selected ten EEG recordings and then filtered using MEMD method as the clean EEG signals as shown in Figure 1(b). Next, we add EOG noise to the clean data with different noise signal ratio (SNR) ranging from 10 dB to −20 dB with a step of −1 dB with respect to the EEG level as the simulated EEG data corrupted with EOG artifact. Figures 1(c) and 1(d) show the simulated EEG data corrupted with two different EOG levels. Secondly, once again ten patients have been selected at random to assess and compare the ability of entropies from each independent scale to distinguish the patients' states under consciousness or anesthesia. So EEG data during preoperation and maintenance stages are collected and filtered as mentioned above. Finally, all 26 patients are used to obtain a new single index reflecting the DOA from MSE via ANN method.

### 2.3. Expert Assessment of Conscious Level

**Figure 2:**
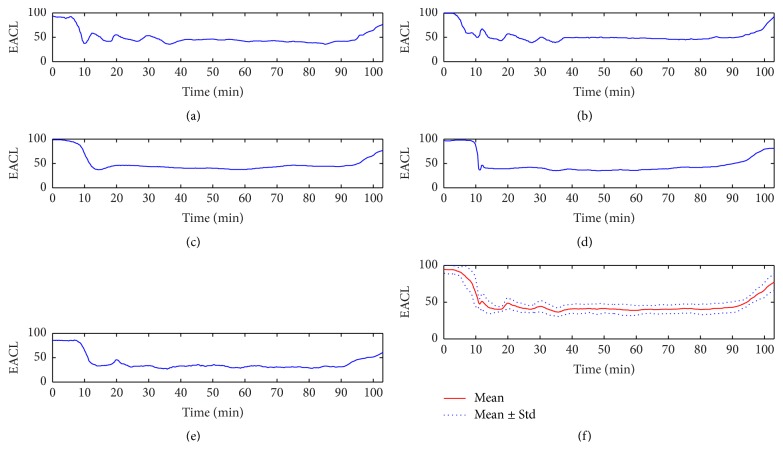
An example of EACL from five doctors. (a) Doctor A. (b) Doctor B. (c) Doctor C. (d) Doctor D. (e) Doctor E. (f) Mean and standard error of EACL from five doctors.

Firstly, two research nurses keep observing the state of patients and recording the events and signs which happen during surgery in operation room and possibly have relationship with “the state of anesthetic depth” in detail and carefully [38], for example, the start and end time of the anesthetic events including induction and extubation, drugs administered time and their dose, MAC values recorded every five minutes during the whole period of anesthesia, and so on. Then, five experienced anesthesiologists need to make decision by the individual to plot the changes of “the state of anesthetic depth” of patients over the whole duration of operation based on anesthesia record and their previous experiences. A continuous curve was provided by each doctor to represent how deep under anesthesia the patient is. In order to be consistent with BIS, the range of these curves is from 0 to 100, and 100 indicates totally awake state and 0 is equivalent to EEG silence, and a value between 40 and 60 represents an appropriate anesthesia level during surgery for general anesthesia. Because the original curve was plotted by hand drawing, so, finally, it is digitalized and resampled with a frequency of 0.2 Hz like BIS index to a single dimensionless number series called expert assessment of conscious level (EACL) [38]. Each anesthesiologist with different experience may have a different perspective on EACL; therefore, in order to measure consciousness level more accurately, the mean values of EACL from five anesthesiologists were obtained as target instead of BIS index.  Figure 2 gives an example of EACL from five doctors. Because these five doctors have worked as anesthesiologists specially trained to give anesthesia for many years, the EACL they plotted based on anesthesia recordings and their experiences could be used as a real gold standard of DOA [38].

### 2.4. Multivariate Empirical Mode Decomposition Based Filter

MEMD proposed by Rehman and Mandic in 2010 [39] is an improved algorithm of empirical mode decomposition (EMD) which was introduced by Huang et al. in 1998 [40]. In EMD method, a signal is decomposed with iterative process into several ordered elements called intrinsic mode functions (IMF) ranging from high to low frequency [41]. In comparison with conventional methods such as Fourier and Wavelet decomposition, the EMD is driven by data adaptively without fixed basis functions. So it is highly suitable for analyzing nonlinear and nonstationary signals. And the original signal *X*(*t*) can be reconstructed by summing up all IMFs as follows:

(1) $$\begin{array}{c} X\left(\;t\;\right)=\overset{N}{\underset{i=1}{\sum }}c_{i}\left(\;t\;\right)+r_{N}\left(\;t\;\right), \end{array}$$

where *N* is the total number of IMFs decomposed by EMD, *c*_*i*_(*t*) is the *i*th IMF, and *r*_*N*_(*t*) is the residue.

The unwanted artifacts or noise can be removed by recomposing the signal with different IMFs according to the following equation:

(2) $$\begin{array}{c} \hat{X}\left(\;t\;\right)=\overset{q}{\underset{p}{\sum }}c_{i}\left(\;t\;\right), \end{array}$$

where $\hat{X}(t)$ is the filtered signal, *c*_*i*_(*t*) is the *i*th IMF as mentioned above, and *p*, *q* ∈ (1, *N*). When 1 < *p* ≤ *q* = *N*, the signal is reconstructed with low frequency elements which means a low pass filter, when 1 = *p* ≤ *q* < *N*, low frequency noise is removed which means a high pass filter, when 1 < *p* ≤ *q* < *N*, it means a band pass filter, and when *p* > *q*, (2) can be expressed as follows:

(3) $$\begin{array}{c} \hat{X}\left(\;t\;\right)=\overset{q}{\underset{1}{\sum }}c_{i}\left(\;t\;\right)+\overset{N}{\underset{p}{\sum }}c_{i}\left(\;t\;\right); \end{array}$$

in this case, it means a band stop filter. According to analysis above, we can remove EOG from EEG signal by combining the selected IMFs which is signal dominated.

**Figure 3:**
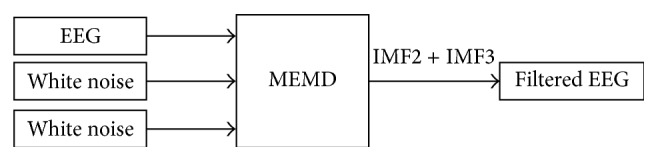
Schematic illustration of MEMD based filter.

Although the MEMD is introduced to decompose multichannel signals, it also can be used in a single channel signal by combination of original signal and independent white noise added into extra channels to form a multivariate signal. By this means MEMD, unlike ensemble empirical mode decomposition (EEMD) which decomposes white noise added signal and then averages out the noise by sufficient number of trials [42], solves the problem of mode mixing to a certain extent caused by EMD without introducing any white noise into original data [39]. In comparison with EEMD, MEMD introduces no noise and consumes less time when decomposing the signals. In this paper, MEMD is applied to remove unwanted signals from EEG. According to the previous study [43], we reconstruct the EEG signals by summing IMF2 and IMF3 after decomposition as the filtered signals as shown in Figure 3.

The conclusion that filtered EEG signals are reconstructed using IMF2 + IMF3 is a statistically based, empirically derived by comparison of all possible combinations of IMFs for discriminating preoperation, induction, maintenance, and recovery stages and tracing the changes of consciousness level in our previous study [43]. Thirty patients' data were collected for statistical analysis. Firstly, according to frequency ranges of the EEG signals, IMF2, IMF3, IMF4, IMF5, and IMF6 were considered for next combination. So there totally are 31 different ways. Then due to the entropy values during anesthesia state are less than awake state, IMF2, IMF2 + IMF3, IMF2 + IMF4, IMF2 + IMF3 + IMF4, and IMF2 + IMF3 + IMF6 were selected for the next analysis. Finally, *p* values of entropy values were calculated to compare the statistic difference between awake and anesthesia state and IMF2 + IMF3 with least *p* value which is also less than 0.05 was used as acceptable filtered EEG.

### 2.5. Sample Entropy and Multiscale Entropy

The SampEn is proposed by Richman and Moorman in 2000 [21] to measure the complexity of physical time series according to the following steps.

For a given time series with *N* points {*X*(*i*), 1 ≤ *i* ≤ *N*}, embed dimension *m*, tolerance *r*.

(1) Form *N* − *m* + 1 vectors *X*_*m*_(*i*) according to the template defined as
  (4) Xmi=xi+1,xi+2,xi+3,…,xi+m−1,1⩽i⩽N−m+1.
(2) Calculate the distance between two different vectors mentioned above as
  (5) dXmi,Xmj=max⁡Xmi+k−Xmj+k,for  0⩽k⩽m−1.
(3) Count the total number of vectors *X*_*m*_(*j*) within *r* of *X*_*m*_(*i*) denoted by *B*_*i*_ and then
  (6) Bimr=1N−m−1Bi,Bmr=1N−m∑i=1N−mBimr.
(4) Set *m* = *m* + 1 and repeat steps (1) to (3):
  (7) $$\begin{array}{c} A^{m}\left(\;r\;\right)=\frac{1}{N-m}\overset{N-m}{\underset{i=1}{\sum }}A_{i}^{m}\left(\;r\;\right). \end{array}$$
(5) Denote the SampEn by
  (8) $$\begin{array}{c} \text{SampEn}\left(\;m,r,N\;\right)=-\ln\frac{A^{m}\left(r\right)}{B^{m}\left(r\right)}. \end{array}$$

Although SampEn is popular and useful in application of measuring complexity of signal, it does not consider the structure information related to time scales. Therefore, Costa et al. proposed MSE algorithm to analyze signals over different scales [25]. Firstly, for a given scale *τ*, a “coarse-graining” process is made by averaging all the data points located in a window which moves with step *τ*, after which we get a new time series

(9) $$\begin{array}{c} y_{j}^{\left(\tau \right)}=\frac{1}{\tau }\overset{j\tau }{\underset{i=\left(j-1\right)\tau +1}{\sum }}X_{i}. \end{array}$$

**Figure 4:**
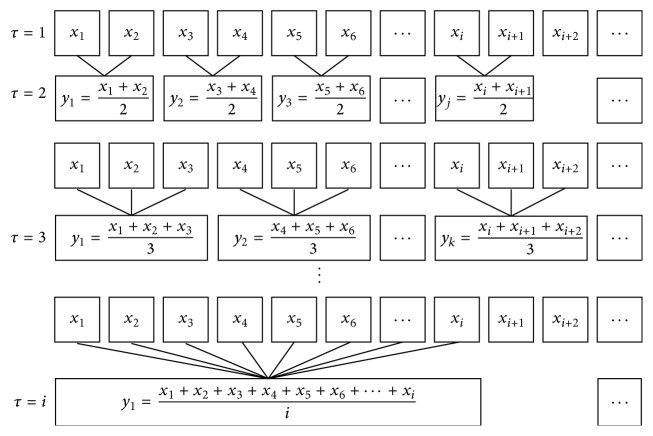
Illustration of the coarse-graining procedure for MSE calculation. Adapted from [25].

**Figure 5:**
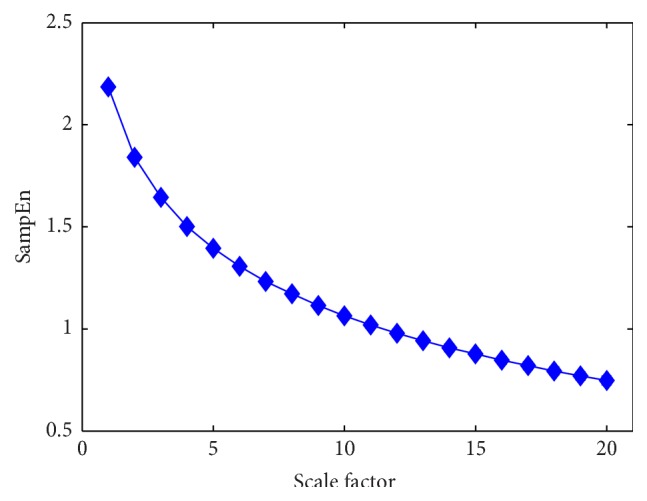
MSE analysis of 30 simulated Gaussian white noise. Each noise contains 30000 data points. Symbols represent mean values of entropy for the 30 time series and error bars the SD. Adapted from [24, 25].

Then SampEn algorithm is used for each new time series after “coarse-graining” process related to time scale *τ*.  Figure 4 shows the flow chart of coarse-graining procedure. And Figure 5 gives an example of MSE calculated from 30 simulated Gaussian white noise.

In this paper, the parameters are set as follows: *τ* = 1,2, 3,4,…, 20, *m* = 2, and *r* = 0.2 according to the statistical analysis of previous studies [21, 25, 30].

### 2.6. Artificial Neural Network

**Figure 6:**
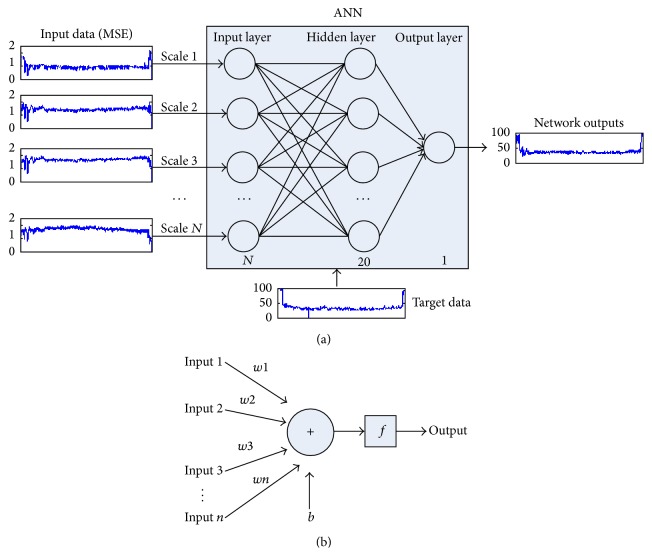
The flowchart and structure of ANN. (a) The flowchart of proposed MSE based method via ANN and the structure of ANN network. (b) The structure of single neuron in ANN for each layer.

In this research, a new method is proposed to obtain an index for monitoring DOA as shown in Figure 6.  Figure 6(a) shows the detailed structure of ANN network and Figure 6(b) illustrates that structure for each neuron. *W* = [*w*1, *w*2, *w*3,…, *wn*] is the weights of each neuron and *b* is its bias. In order to consider the structure information related to multiple scales, we measure the complexity of EEG by MSE analysis. Then the multiple scale entropies are transformed into a single index using nonlinear regression method (e.g., ANN) to build the functions between MSE and the gold standard. Generally, an integrated ANN model contains input layer, hidden layer, and output layer. In this paper, the input layer consists of *N* neurons ranged from 1 to 20 consistent with the number of inputs, hidden layer contains 20 neurons, and output layer has 1 neuron, respectively. The target data is one-dimensional series regardless of the number of inputs. We choose feed-forward backpropagation which is a very common method to train ANN model as the learning rule. Then the entropies of all time scales calculated from EEG and gold standard are treated as the input data and target data to train, validate, and test the ANN model. There are multiple inputs to this network and 1 output. In order to confirm the performance of the new combined index, we also compare it with the entropy results related to a single scale from scale 1 to scale 20 via ANN. In this situation, the input data of ANN is entropy values from a single scale. Furthermore, the samples percentages divided randomly for training, validation, and testing are 70%, 15%, and 15%, respectively. All analyses were performed in MATLAB (v7.13, MathWorks Inc., USA).

## 3. Results

In this section, we compared the sensitivity of all entropy indexes of each independent time scale from MSE analysis of simulated EEG corrupted with different level EOG artifact. And then we analyzed the ability of each single scale entropy to distinguish the consciousness and anesthesia states of patient during surgery. Finally, the proposed method is applied to real EEG signals collected from patients.

### 3.1. Sensitivity of Single Scale Entropy to EOG

In this section, the signals are used to evaluate the sensitivity of single scale entropy to EOG artifacts. The target of the filtering is to remove artifacts in EEG signals. Through adding EOG noise, we simulated the contaminated EEG with different noise level; then coefficient variation (i.e., the ratio of the standard deviation to the mean, CV) of MSE at each time scale is statistically analyzed to compare their robustness to noise.

**Figure 7:**
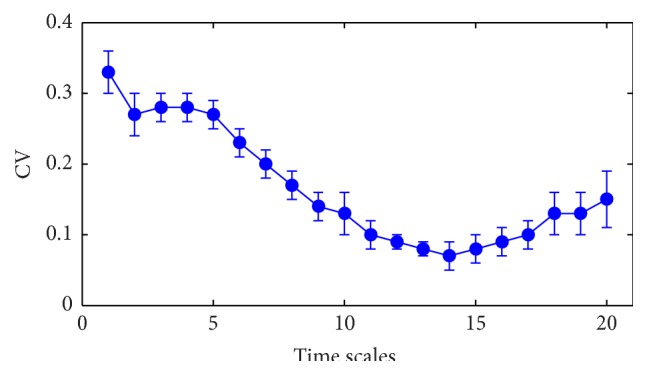
The mean and standard deviation of CV of entropy values for 32 different levels' EOG noise. Symbols represent mean values of CV from ten cases with different level of noise and error bars the SD.

The EEG signals collected from ten patients under preoperation are used after filtration by summing IMF2 and IMF3 based on MEMD algorithm [43]. Then the EOG as the simulated artifact is added into the filtered EEG of each case with different SNR ranging from 10 dB to −20 dB with the step of −1 dB. Considering the original filtered EEG, there is 32 different levels' signal plus the original filtered EEG for each case. A sliding window with 30-second length including 3750 data points is utilized when measuring the complexity of EEG signals using MSE analysis and moves forward once every five seconds for real time DOA monitoring. The CV of the entropy index for each single scale to the EOG artifact are analyzed. We also plot the mean and standard deviation for ten cases as indicated in Figure 7. We can see that the CV decreases with the increasing of scales until scale 14 and then it rises slightly but extremely less than the value of scale 1. The results indicate that the entropy at scale 1 is the most sensitive to EOG artifact. The possible reason is that entropy values on small time scales mainly represent the information of high frequency parts. And values on large time scales indicate the low frequency information due to the “coarse-graining” process which averages the data points within a fixed-size window so that the high frequency parts are removed from original signals [24, 32]. With the “coarse-graining” process, the amplitudes of EOG decrease, so EOG have less effect on EEG signals. It is noted that MSE at scale 1 are the most sensitive to EOG artifact, which means that SampEn is prone to artifacts. So combination of MSE at multiple time scale instead of SampEn may provide a more robust method to monitor DOA.

### 3.2. Comparative Study of Each Scale for Distinguishing Different States

**Figure 8:**
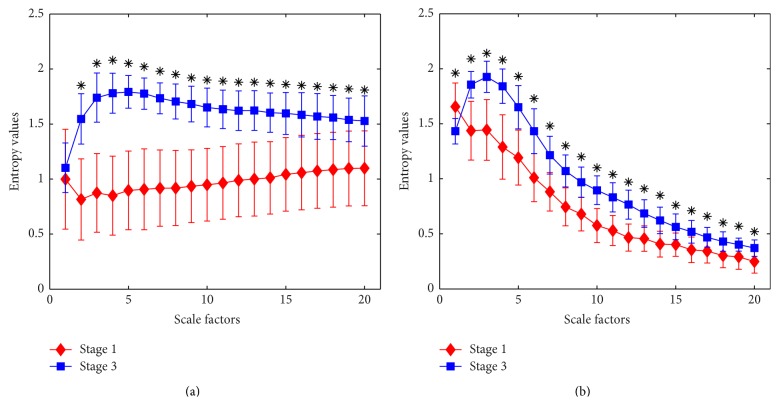
Statistical analysis of MSE from ten patients' EEG under different states at multiple time scales. Stage 1 is preoperation and stage 3 is maintenance during surgery. Symbols marked with “*∗*” indicate significant difference between stage 1 and stage 3 with *p* < 0.05. (a) Before filtering. (b) After filtering.

In order to further evaluate the ability of MSE at different time scales to distinguish the patients' states during surgery, we test the MSE on real EEG signals collected from ten patients under preoperation and maintenance stages before filtering. Moreover, we investigate the effect of filtering by summing IMF2 and IMF3 [43] on MSE at each independent scale. As shown in Figure 8, the error bar at each scale represents the mean and standard deviation of an entropy measured from ten patients. For time scale one, the mean value of entropy is very close between preoperation and maintenance before filtering which indicates that it is difficult to differentiate these two stages using MSE at scale factor of one. With the increasing of time scales, the entropy values from stage 1 decrease significantly and then rise slightly, but values from stage 3 ascend extremely and then decline slowly.

Then the paired-samples two-tailed *t*-test was used to compare the difference of MSE from ten patients' EEG between stage 1 and stage 3 at multiple time scales before and after filtering. The level of significance was set at *p* < 0.05 as shown in Figure 8. At all scales except scale 1, the means of the entropy values have significant difference between these two stages before filtering. The second figure shows the results from analysis after filtering; the means of the entropy values at all scales are statistically significantly different. The filter performs usefully at scale 1. Although the difference of entropy between stage 1 and stage 3 decreases at large time scales regardless of being filtered or not, MSE at large scales also have the capability of differentiating stage 1 and stage 3 with *p* < 0.05. We can see that the entropy value from stage 1 is largely outweighing the value from stage 3 at the scale of one which means that EEG from patients under consciousness perform with more complexity than under anesthesia state. Therefore, entropy at scale 1 can distinguish the EEG collected from patients under these two stages after filtering with *p* < 0.05. It is consistent with SampEn which is equal to the MSE at a time scale of one to monitor DOA during surgery according to previous researches [22, 30, 43]. And then the entropy values from stage 3 surpass stage 1 which means that EEG from patients under maintenance state are more complexity in comparison with preoperation at large time scale.

The entropy values reduce greatly for both stages in Figure 8(b) compared to Figure 8(a) when scale factor exceeds 3. It is possible because low frequency parts are removed from original EEG signals by summing IMF2 and IMF3, so entropy values at large scales related to low frequency elements decrease a lot. And filtering also changes the SD of time series which will affect the tolerance level. Because main element reserved, SD remain largely unchanged compared to prefiltering in spite of slight decrease, while, with the loss of high frequency elements due to coarse-graining procedure, the amplitude decreases. Therefore, slight change in the tolerance level compared with relative lower amplitude indicates that fewer vectors will be distinguishable and that complexity of signal will decrease [24]. When comparing these two figures, the entropy value from stage 1 after filtering is larger than before filtering at time scale 1 because EOG artifacts which are of low frequency and relatively less complexity are canceled from EEG. The entropy at scale 1 is extremely sensitive to EOG artifact.

By the above analysis, entropy at all scales can make a contribution to discriminate the EEG during preoperation and maintenance stages although the ability of MSE at some scales is very weak no matter before or after filtering. And it is not robust to monitor DOA using SampEn which is equal to MSE at scale 1.

### 3.3. Performance Evaluation for Monitoring DOA

In this section, 26 patients' EEG signals collected during surgery are used to investigate the performance of our proposed method to monitor DOA. A sliding window with 30-second length including 3750 data points is utilized when measuring the complexity of EEG signals using MSE analysis and moves forward once every five seconds for real time DOA monitoring. The prediction of ANN is quantified by coefficient of determination, denoted by *R* ^2^; a measure of the proportion of total variation of network outputs is replicated by ANN model. *R* ^2^ is larger when prediction value of ANN is closer to target data. Furthermore, correlation coefficient (Corrcoef) is employed to measure the linear correlation between the new index obtained from MSE via ANN and gold standard to confirm the accuracy and robustness of proposed method for DOA monitoring.

**Figure 9:**
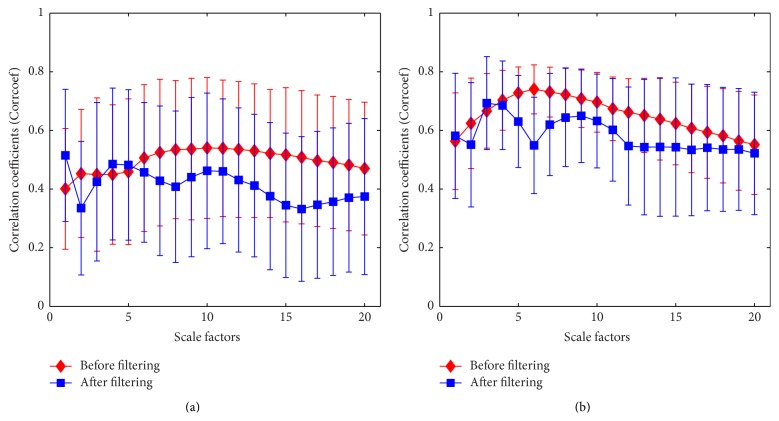
Correlation coefficients of MSE and gold standard at different time scale before and after filtering. Symbols represent mean values of correlation coefficients between MSE based measurement and gold standard from 26 cases and error bars the SD. (a) BIS index was used as gold standard. (b) EACL was used as gold standard.

As shown in Figure 9, there presents a moderate linear relationship between MSE at each scale from 1 to 20 and gold standard before filtering and a moderate or even weak linear relationship after filtering. The correlation coefficients produced at large scales appear to be consistent with the analysis mentioned above which indicates that the MSE at large scales are less capable of tracking the consciousness level of the patients. The *R* values of ANN model are relatively low; since the relationship between MSE at single scales and gold standard is not so strong, the ANN model misses entropy points by much. Furthermore, the correlation coefficient at scale 1 after filtering is higher than before filtering. It demonstrates that the filtering algorithm used in this study is most effective for MSE at scale 1 (i.e., SampEn) as indicated in Figure 9 consisting of the error bars which represent the mean and standard deviation but remove lots of information related to large scales. Therefore, the mean and standard deviation of correlation coefficient and *R* value after filtering are smaller in comparison with those before filtering. And the large values of standard deviation in Figure 9 suggest that MSE at single scales is extremely sensitive to noise for monitoring DOA.

Tables 1 and 2 show results focused on combinations by changing scales from 1 to 20 from MSE of EEG to form a composite indicator for measuring DOA before and after filtering. For example, 1-1 representing the input data of ANN model is MSE at only time scale 1, and 1–20 indicates that there are 20 scales from 1 to 20 used to train the ANN model as inputs and so on. We can note that both *R* values and correlation coefficient increase generally in spite of some fluctuation with adding more entropies at different scales into the network as inputs. Indeed, by the above analysis, the MSE at different scales make contribution to track the anesthesia level from EEG analysis. So combination of multiple scales can enhance this feature and perform better to measure the DOA than single scale. Furthermore, it is indicated that CV decrease with adding more scales. The lower CV value suggests that the corresponding index performs less sensitive to noise, because the means of the entropy values at scale factor 2–20 are statistically significantly different no matter before or after filtering as shown in Figure 8. So it is uncertain that the nonfiltering produces better results than filtering if scale factors 2–14 are selected. But it is confirmed that if all scales ranged from 1 to 20 are used, there will be better results. MSE at scale from 2 to 20 before and after filtering have similar performance but worse than MSE at scale from 1 to 20 as shown in Tables 1 and 2. Furthermore, the similar performance between before and after filtering indicates that MSE based index via ANN is robust to noise.

Based on the results above, entropies at all scales from 1 to 20 are used to train ANN model as input data to obtain a composite indicator for DOA monitoring. Tables 3 and 4 show the results of 26 patients using our proposed method to monitor DOA using BIS and EACL as target during surgery. MSE based index via ANN appears to be a very strong positive correlation with the gold standard and thus performs extremely better compared with SampEn for monitoring DOA during surgery. Moreover, it is evident in Figure 9 that the correlation between MSE at scale factors of 1, 4, and 5 and BIS is higher after filtering than that before filtering; thus, a feasible optimization method is built by selecting entropies at which scales perform better before and after filtering to retrain the ANN model and acquire a new index listed in the final column in Table 3. That is to say, entropies at the scale factors of 1, 4, and 5 after filtering and other factors before filtering are used to train the ANN as inputs. The correlation is higher between MSE from combination of pre- and postfiltering and BIS compared with SampEn and MSE from pre- and postfiltering, respectively. Besides, CV of MSE from combination of pre- and postfiltering (i.e., 9.64) is extremely smaller than SampEn (i.e., 52.50 and 45.10) and MSE (i.e., 16.00 and 20.00) before and after filtering, respectively. It indicates that optimization method performs more accurate robustness to monitor the DOA as a new indicator.

Tables 3 and 4 show the statistic results of MSE based measurement via ANN with EACL and BIS as gold standard. The proposed MSE based method not only has high correlation with BIS index but also is very similar to EACL. It indicates that this method is successful in measuring the consciousness level and monitoring DOA. Furthermore, the proposed method using EACL as target performs more accurately with higher correlation compared with BIS. It is known that BIS is prone to artifacts, while the proposed method based on MSE and ANN is extremely robust due to the high similarity to gold standard (i.e., EACL and BIS) no matter before or after filtering. Moreover, BIS index have been questioned for its reliability to monitor DOA, so using EACL as gold standard would be more acceptable.

Furthermore, in this paper, 10 patients received desflurane, 13 patients received sevoflurane, and 3 patients received propofol as anesthesia agents.  Table 5 presents the mean and standard deviation of correlation coefficient between MSE via ANN and EACL for monitoring DOA in terms of propofol, sevoflurane, and desflurane, respectively. There is a high correlation for each agent, especially MSE via ANN by combination of pre- and postfiltering. Therefore, there is no difference between propofol, sevoflurane, and desflurane for DOA monitoring.

## 4. Discussion

SampEn is a method widely used in many researches to measure complexity of signals and monitor DOA during surgery [21, 22, 43]. MSE, an improved algorithm from SampEn, measures complexity of signal at different time scales and is also commonly applied to complex physiological time series [24, 25, 29, 30, 32]. However, a single index needed from the MSE analysis for monitoring DOA and relative complexity at multiple scales must be taken into account in clinical applications. ANN which can adaptively and optimally evaluate the function between MSE and a single index depending on the input and target data by training, validating, and testing provide a special solution to this task. When they were taken into account independently, all the scales are analyzed to confirm the sensitivity to noise and contribution to strengthen the indicator's preciseness for prediction DOA.

EOG artifact which is the most common noise in EEG is added to original signals with different level. The results show that MSE at the scale factor of one is more sensitive to noise with high CV which is also proved in 26 patients' real EEG signals. MSE are calculated using SampEn algorithm after coarse-graining procedure. The accuracy of SampEn depends on time series length [21]. The discrepancies between SampEn values numerically increase with the decrease of data length [24], while this coarse-graining procedure decreases the number of data points with the increase of time scale. Although it is uncertain of the minimum length of data required to calculate MSE, the error because of decreased data number will increase [24]. In this paper, the window size for MSE calculation is 3750. There are less than 250 data points when scale factors are more than 15. The consistency of SampEn is extremely decreases. So CV increases again in the last few scale factor as shown in Figure 7.

And then we investigate the ability to distinguish and track the change of anesthetic states; MSE at scale 1 perform better after filtering than before filtering, but filtering algorithm removes lots of information associated with large time scales in spite of filtering out physiological and external noise effectively by summing IMF2 and IMF3 [43]. We also note that filtering takes no effect on proposed method when measuring anesthesia depth as shown in Tables 3 and 4. The reason is that the ability of MSE at scale factor of one to distinguish patients' states increases after filtering in spite of decrease at larger scales in comparison with prefiltering as indicated in Figure 8. And standard deviations of MSE for stage 1 at each scale after filtering are smaller than those before filtering. Therefore, the filtering takes little effect on overall performance of proposed approach for monitoring DOA. Finally in order to optimize the composite index, the entropies at which scales perform better before and after filtering are selected to train ANN model. The correlation between MSE from combination of pre- and postfiltering and the gold standard is highest compared with SampEn and MSE from pre- and postfiltering, respectively. The results confirm that our proposed method is more accurate and robust to measuring DOA than SampEn.

Generally, the frequency of EEG signal can be divided into bands, and the pattern within a certain frequency range contains the corresponding biomedical features. So, EEG filtered with different passed band have different useful characteristics for monitoring DOA. Entropy monitoring commercially developed by Datex-Ohmeda measures DOA of patient at two different frequency bands which produces response entropy and state entropy. The combination of different parameters derived from multiple bands of EEG would provide more accurate knowledge for DOA monitoring. SampEn measures the complexity of EEG at single time scale. However, MSE measures the complexity of times series at different time scales. The features enhanced by filter used for optimizing SampEn may not be suitable for MSE at different scales. It is noted that there is a significant difference between stage 1 and stage 3 after filtering for MSE at scale 1, but there is no difference before filtering as shown in Figure 8. So it is reasonable to consider MSE at scale 1 after filtering instead of before filtering as one of the inputs to obtain complex parameter via ANN. For MSE at other scales, though there is a significant difference between stage 1 and stage 3 no matter before or after filtering, the correlation after filtering between MSE at scale 3 for EACL and scales 4 and 5 for BIS as target is higher than before filtering, so MSE with better performance are chose to measure DOA. In this way, the complex index derived from MSE at multiple time scale with better performance via ANN would be more accurate to characterize DOA. In this paper, the filtered EEG reconstructed by IMF2 + IMF3 are used to optimize MSE at scale 1. If reconstructed using different combination of IMFs to optimize MSE at every other scale, we combine all optimizing MSE at corresponding scale via ANN; then the index calculated by proposed method would be more accurate for DOA monitoring. It is our next work in the future to find the combination of IMFs to optimize MSE at every other scale.

In conventional methods, time and frequency domains analyses of EEG signals are used to measure the consciousness level such as median frequency, spectral edge frequency, spectral entropy, and ApEn. ApEn is a valuable method to calculated complexity from a dynamical system in phase space. Because ApEn sets a threshold for noise cancellation, it is better than conventional method in measuring the consciousness level from EEG recordings [20, 44]. Furthermore, SampEn is the improved algorithm from ApEn so it performs better than or as good as ApEn at least [21, 22]. The combined index behaves better than SampEn as reference performance measure confirmed by higher correlation with gold standard. In our opinion, it is adequate to demonstrate the performance of present index in this paper. Nevertheless, there are many new methods applied to EEG signals to measure nonlinear and complexity. Gifani et al. proposed optimal fractal-scaling analysis to quantify human EEG dynamic for depth of anesthesia [45]. In 2012, Kumar et al. used fractal dimension of EEG to assess hypnosis state of patient during anesthesia [46]. And, in 2015, Hayashi et al. used poincaré analysis in estimating anesthesia depth [47], and Melia et al. provided a methodology for prediction of nociceptive responses during sedation to quantify analgesia level from EEG signals in high frequencies [48]. They were successfully applied in measuring the depth of anesthesia based on EEG signal in special aspect. Future work is needed to draw the conclusion whether our method performs better than these methods mentioned above in detail. It is difficult to say which method is best [49]; on the one hand, it is impossible to apply all these methods to the current population under study. And each method also can use different parameters and improved algorithms. For example, a refined version of the MSE may further improve the performance to a certain extent. A detailed study of implication using different MSE methods will be demonstrated in future. On the other hand, these methods may be tested under different condition and database. It is worth having an attempt to fuse multiple parameters extracted from EEG for a reliable monitor.

In addition, during deep anesthesia, burst suppression in EEG is recognised as light anesthesia which is a serious problem in EEG based indicators when other methods are used such as median frequency and spectral edge frequency [20, 44]. BIS can successfully avoid this problem by definition of a burst suppression ratio [13]. In present study, there is no burst suppression component in collected EEG signals; thus, this problem has no effect on our results. Furthermore, ApEn and SampEn can correctly monitor burst suppression occurring during deep anesthesia as anesthesia concentration increases according to previous studies [20, 21, 44]. MSE as an advanced method improved from SampEn could calculate the complexity of data series over different time scales. It is reasonable to believe that EEG analysis using MSE for DOA monitoring via regression with BIS can avoid misclassification of burst suppression although more future work is needed for confirmation.

Propofol, sevoflurane, and desflurane are three commonly used anesthesia agents for induction and maintenance of general anesthesia. They have been known to have the same mechanisms of action, all through potentiation of GABA_A_ receptor activity [50]. EEG research finds that they cause a prominent decrease in gamma-band activity undergoing general anesthesia [51]. Propofol may be the preferred induction anesthetic for a shorter time surgery compared with sevoflurane or desflurane [52]. However, there are many previous studies to show that propofol does not show a significant difference compared with sevoflurane or desflurane in patients undergoing surgery for anesthetic induction and maintenance [52, 53]. It is consistent with our results shown in Table 5. However, more data is needed to confirm this conclusion in the future.

The purpose of this paper is to propose a new approach using MSE via ANN and confirm its performance for monitoring DOA. The parameters of ANN such as the neurons number of each layer in ANN model seem to have less limitation on the results, so the parameters are selected regardless of optimization. Nevertheless, we will try to optimize all the parameters as far as possible in the next step for application to practice. We note that MSE at large scales can make contribution to track the change of consciousness level of patients in spite of being very weak and the correlation between composite index based on MSE via ANN and gold standard strengthens with adding more scales to train the model. However, the maximum scale of MSE is set to be 20 in this study. So more experiments are need to confirm the effect of scales larger than 20. Furthermore, in order to integrate MSE over different scales into a single index, we need to select the appropriate scales. In this paper, we analyze parts of various combinations and integrate MSE at all scales into the single indicator of anesthesia depth. More deliberate selection of scale combinations are needed to be further explored.

The EACL data are derived from five experienced anesthesiologists through quantifying the consciousness levels of each patients according to operation recordings and their experience as the depth of anesthesia. By this means, the present method can avoid the problems occurring in BIS and thus can be extended to other anesthesia techniques. However, this mentioned method measures consciousness level based on EEG signals generated by cerebral cortex like BIS; not all drugs administered for anesthesia act on this part. For example, if they are acting on thalamus and brain stem [54], this method is not suitable in these cases.

## 5. Conclusions

In this paper, a new method is proposed to monitor DOA of patients during surgery based on MSE via ANN. Its effectiveness is evaluated by correlation analysis with BIS. The new index performs extremely better than the raw single scale MSE index and SampEn. The index from MSE by combination of pre- and postfiltering is the most accurate indicator for determining the DOA in patients during surgery. There is a very strong positive correlation (i.e., 0.83 ± 0.08) between proposed index and BIS and a lower CV (i.e., 9.64%) which indicates that the new approach can be very useful for accurate and robust measurement of DOA.

## Figures and Tables

**Table 1:** Different combinations of multiple scales changing from 1 to 20 via ANN for monitoring DOA using BIS as gold standard. 1-1 in the first column represents the input data of ANN model that is the entropy at time scale 1, and 1–20 indicates that there are multiple scales from 1 to 20 and so on.

Scale(s)	Before filtering			After filtering		
	*R*	Corrcoef	CV (%)	*R*	Corrcoef	CV (%)
1-1	0.44	0.40 ± 0.21	52.50	0.53	0.51 ± 0.23	45.10
1-2	0.47	0.50 ± 0.27	54.00	0.68	0.70 ± 0.19	27.14
1–3	0.67	0.68 ± 0.19	27.94	0.69	0.70 ± 0.18	25.71
1–4	0.69	0.70 ± 0.14	20.00	0.72	0.72 ± 0.15	20.83
1–5	0.69	0.70 ± 0.14	20.00	0.72	0.72 ± 0.15	20.83
1–6	0.71	0.71 ± 0.14	19.72	0.73	0.73 ± 0.15	20.55
1–7	0.71	0.71 ± 0.14	19.72	0.73	0.73 ± 0.14	19.18
1–8	0.71	0.71 ± 0.14	19.72	0.74	0.73 ± 0.14	19.18
1–9	0.72	0.72 ± 0.14	19.44	0.75	0.74 ± 0.14	18.92
1–10	0.71	0.71 ± 0.16	22.54	0.75	0.74 ± 0.14	18.92
1–11	0.72	0.72 ± 0.14	19.44	0.76	0.75 ± 0.13	17.33
1–12	0.75	0.74 ± 0.12	16.22	0.76	0.75 ± 0.14	18.67
1–13	0.73	0.73 ± 0.14	19.18	0.76	0.75 ± 0.13	17.33
1–14	0.76	0.74 ± 0.12	16.22	0.76	0.75 ± 0.13	17.33
1–15	0.75	0.74 ± 0.13	17.57	0.76	0.74 ± 0.14	18.92
1–16	0.75	0.74 ± 0.13	17.57	0.76	0.75 ± 0.13	17.33
1–17	0.76	0.74 ± 0.13	17.57	0.77	0.75 ± 0.13	17.33
1–18	0.76	0.74 ± 0.13	17.57	0.75	0.75 ± 0.14	18.67
1–19	0.76	0.74 ± 0.13	17.57	0.76	0.75 ± 0.12	16.00
1–20	0.76	0.75 ± 0.12	16.00	0.76	0.75 ± 0.15	20.00
2–20	0.74	0.73 ± 0.13	17.81	0.73	0.72 ± 0.13	18.06
Mean ± std	0.70 ± 0.09	0.70 ± 0.09	22.30 ± 10.61	0.73 ± 0.05	0.73 ± 0.05	20.63 ± 6.21

**Table 2:** Different combinations of multiple scales changing from 1 to 20 via ANN for monitoring DOA using EACL as gold standard. 1-1 in the first column represents the input data of ANN model that is the entropy at time scale 1, and 1–20 indicates that there are multiple scales from 1 to 20 and so on.

Scale(s)	Before filtering			After filtering		
	*R*	Corrcoef	CV (%)	*R*	Corrcoef	CV (%)
1-1	0.56	0.56 ± 0.16	28.57	0.61	0.58 ± 0.21	36.21
1-2	0.80	0.80 ± 0.07	8.75	0.82	0.83 ± 0.05	6.02
1–3	0.81	0.82 ± 0.06	7.32	0.82	0.83 ± 0.05	6.02
1–4	0.82	0.82 ± 0.07	8.54	0.84	0.85 ± 0.05	5.88
1–5	0.81	0.81 ± 0.06	7.41	0.84	0.84 ± 0.05	5.95
1–6	0.81	0.82 ± 0.07	8.54	0.84	0.85 ± 0.05	5.88
1–7	0.83	0.83 ± 0.06	7.23	0.85	0.85 ± 0.04	4.71
1–8	0.83	0.83 ± 0.06	7.23	0.84	0.85 ± 0.04	4.71
1–9	0.83	0.83 ± 0.06	7.23	0.85	0.85 ± 0.04	4.71
1–10	0.84	0.84 ± 0.05	5.95	0.84	0.84 ± 0.05	5.95
1–11	0.84	0.84 ± 0.05	5.95	0.85	0.85 ± 0.04	4.71
1–12	0.84	0.84 ± 0.05	5.95	0.85	0.86 ± 0.04	4.65
1–13	0.83	0.83 ± 0.06	7.23	0.86	0.86 ± 0.04	4.65
1–14	0.85	0.85 ± 0.05	5.88	0.86	0.86 ± 0.04	4.65
1–15	0.84	0.84 ± 0.05	5.95	0.86	0.86 ± 0.04	4.65
1–16	0.85	0.85 ± 0.05	5.88	0.86	0.86 ± 0.04	4.65
1–17	0.85	0.85 ± 0.05	5.88	0.86	0.86 ± 0.04	4.65
1–18	0.86	0.85 ± 0.05	5.88	0.86	0.87 ± 0.03	3.45
1–19	0.86	0.85 ± 0.05	5.88	0.86	0.86 ± 0.04	4.65
1–20	0.86	0.85 ± 0.05	5.88	0.86	0.86 ± 0.04	4.65
2–20	0.82	0.82 ± 0.06	7.32	0.81	0.81 ± 0.07	8.64
Mean ± std	0.82 ± 0.06	0.82 ± 0.06	7.83 ± 4.85	0.84 ± 0.05	0.84 ± 0.06	6.67 ± 6.85

**Table 3:** Combination of all scales ranging from 1 to 20 via ANN for monitoring DOA using BIS as gold standard. SampEn as shown in 2th and 4th columns are the correlation coefficients between ANN outputs and BIS using MSE at the scale factor of one as training inputs before and after filtering. The 3th and 5th columns are the results using MSE at scales from 1 to 20 as training inputs before and after filtering and the final column is the corresponding results using MSE at scales 2, 3, and 6–20 before filtering and 1, 4, and 5 after filtering.

Cases	Before filtering		After filtering		Combination of pre- and postfiltering
	SampEn	MSE	SampEn	MSE	
1	0.74	0.87	0.92	0.90	0.92
2	0.38	0.71	0.47	0.70	0.86
3	0.65	0.90	0.51	0.90	0.92
4	0.43	0.86	0.28	0.85	0.92
5	0.85	0.87	0.84	0.91	0.90
6	0.42	0.70	0.31	0.71	0.74
7	0.49	0.73	0.51	0.70	0.77
8	0.12	0.64	0.51	0.68	0.79
9	0.48	0.83	0.74	0.82	0.91
10	0.58	0.73	0.71	0.77	0.81
11	0.00	0.79	0.34	0.85	0.85
12	0.52	0.86	0.75	0.83	0.90
13	0.44	0.85	0.68	0.85	0.90
14	0.22	0.40	0.35	0.37	0.72
15	0.58	0.87	0.67	0.84	0.91
16	0.35	0.78	0.70	0.86	0.85
17	0.28	0.68	0.68	0.75	0.79
18	0.55	0.87	0.18	0.82	0.87
19	0.19	0.79	0.67	0.82	0.84
20	0.45	0.70	0.58	0.75	0.75
21	0.31	0.68	0.49	0.78	0.81
22	0.39	0.70	0.27	0.72	0.76
23	0.32	0.51	0.32	0.33	0.62
24	−0.01	0.56	−0.03	0.53	0.70
25	0.21	0.75	0.59	0.72	0.85
26	0.50	0.83	0.32	0.82	0.83
Mean ± std	0.40 ± 0.21	0.75 ± 0.12	0.51 ± 0.23	0.75 ± 0.15	0.83 ± 0.08
CV (%)	52.50	16.00	45.10	20.00	9.64
*R*	0.44	0.76	0.53	0.76	0.85

**Table 4:** Combination of all scales ranging from 1 to 20 via ANN for monitoring DOA using EACL as gold standard. SampEn as shown in 2th and 4th columns are the correlation coefficients between ANN outputs and EACL using MSE at the scale factor of one as training inputs before and after filtering. The 3th and 5th columns are the results using MSE at scales from 1 to 20 as training inputs before and after filtering and the final column is the corresponding results using MSE at scales 2 and 4–20 before filtering and 1 and 3 after filtering.

Cases	Before filtering		After filtering		Combination of pre- and postfiltering
	SampEn	MSE	SampEn	MSE	
1	0.60	0.85	0.80	0.89	0.88
2	0.64	0.81	0.47	0.84	0.85
3	0.73	0.88	0.24	0.86	0.89
4	0.69	0.92	0.20	0.90	0.93
5	0.83	0.90	0.88	0.91	0.93
6	0.48	0.87	0.60	0.87	0.89
7	0.54	0.90	0.66	0.90	0.91
8	0.36	0.81	0.52	0.82	0.86
9	0.42	0.85	0.56	0.81	0.91
10	0.41	0.78	0.53	0.85	0.80
11	0.19	0.78	0.23	0.85	0.88
12	0.52	0.85	0.76	0.84	0.89
13	0.61	0.90	0.78	0.90	0.91
14	0.42	0.81	0.61	0.83	0.86
15	0.57	0.87	0.70	0.86	0.93
16	0.45	0.80	0.73	0.85	0.89
17	0.32	0.71	0.67	0.79	0.82
18	0.56	0.87	0.62	0.85	0.89
19	0.23	0.81	0.70	0.84	0.87
20	0.43	0.75	0.60	0.74	0.79
21	0.42	0.73	0.51	0.81	0.85
22	0.33	0.74	0.45	0.76	0.82
23	0.42	0.78	0.70	0.79	0.85
24	0.73	0.85	0.74	0.83	0.88
25	0.48	0.84	0.67	0.87	0.89
26	0.66	0.85	0.66	0.89	0.90
Mean ± std	0.50 ± 0.16	0.83 ± 0.06	0.60 ± 0.17	0.84 ± 0.04	0.88 ± 0.04
CV (%)	31.40	6.89	28.83	5.18	4.29
*R*	0.52	0.84	0.61	0.84	0.89

**Table 5:** The statistic results of performance on three different anesthesia agents.

Anesthesia drugs	MSE via ANN before filtering	MSE via ANN after filtering	Combination of pre- and postfiltering
Desflurane	0.82 ± 0.05	0.84 ± 0.03	0.87 ± 0.02
Sevoflurane	0.83 ± 0.05	0.85 ± 0.03	0.88 ± 0.05
Propofol	0.86 ± 0.03	0.84 ± 0.04	0.90 ± 0.02
